# Impact of Body Fat Distribution and Insulin Sensitivity on In Vitro Fertilization Outcomes: A Prospective Observational Study

**DOI:** 10.3390/jcm14113848

**Published:** 2025-05-30

**Authors:** Andrea Roberto Carosso, Alberto Revelli, Alessandro Ruffa, Marco Carosso, Gianvito Contangelo, Chiara Benedetto, Gianluca Gennarelli

**Affiliations:** 1 Obstetrics and Gynecology 1U, Physiopathology of Reproduction and IVF Unit, Department of Surgical Sciences, Sant’Anna Hospital, University of Torino, 10126 Turin, Italy; acarosso@cittadellasalute.to.it (A.R.C.); alessandro.ruffa92@gmail.com (A.R.); gianvito.contangelo@unito.it (G.C.); chiara.benedetto@unito.it (C.B.); gennarelligl@gmail.com (G.G.); 2 Obstetrics and Gynecology 2U, Department of Surgical Sciences, Sant‘Anna Hospital, University of Torino, 10126 Turin, Italy; alberto.revelli@unito.it

**Keywords:** anthropometric measures, infertility, in vitro fertilization, body mass index, waist circumference, hip circumference

## Abstract

**Background:** Since overweight is increasing worldwide, the interest in its potential impact on fertility treatment has increased. Whilst the body mass index (BMI)-based overweight classification is simple, BMI cannot measure body fat distribution. In this research, we aim to investigate whether waist circumference (WC) and waist-to-hip ratio (WHR) are better predictors of ovarian response in IVF cycles than BMI. **Methods:** This prospective observational study included 265 couples undergoing their first IVF/ICSI treatment. BMI, WC, WHR, and insulin sensitivity (measured with homeostatic model assessment (HOMA) index) were assessed at enrollment. The primary outcome of the study was the correlations between the study variables and the ovarian sensitivity index (OSI), calculated according to the formula [(number of retrieved oocytes/total gonadotropin dose) × 1000]. Secondary outcomes were other IVF-related outcomes, including live birth rates. **Results:** The study included 265 women with a mean age of 35.8 ± 4.4 years. The mean BMI was 24.0 ± 4.2 kg/m^2^, WC was 79.1 ± 10.8 cm, and WHR was 0.85 ± 0.09. WC was >80 cm in 102 women and ≤80 cm in 163; WHR was >0.85 in 146 women and ≤0.85 in 119. Higher WC and WHR were both significantly associated with lower OSI, independent of BMI. OSI was lower in women with a WC of >80 cm vs. ≤80 cm (3.2 ± 2.5 vs. 4.6 ± 3.9, *p* < 0.05) and in those with a WHR of >0.85 vs. ≤0.85 (3.4 ± 2.3 vs. 4.9 ± 4.1, *p* < 0.05). Live birth rates did not differ between groups. **Conclusions:** The type of body fat distribution is associated with the ovarian response to controlled ovarian stimulation. In particular, upper body fat correlates negatively with ovarian sensitivity to exogenous gonadotropins. However, potential effects on live birth rates do not seem to be clinically relevant.

## 1. Background

As overweight and obesity are steadily increasing worldwide [1], interest in the potential impact of such conditions on fertility and fertility treatment has increased over the past decades. Indeed, excess body mass seems to exert a negative impact on several aspects of female reproduction [2]. Similarly, overweight and obesity are suggested to have detrimental effects on the outcome of assisted reproductive technologies (ARTs), being associated with decreased live birth rates [3]. Impaired responsiveness to ovarian stimulation, requiring higher doses of gonadotropins; increased duration of stimulation; poorer oocyte retrieval; and increased risk of cycle cancellation are frequently reported in women with obesity [2]. Although the potential impact on oocyte quality has been, and still is, a matter of debate, several recent studies have reported no association between obesity and the proportion of euploid embryos following preimplantation genetic testing (PGT-A) [4,5,6,7]. Nevertheless, higher rates of miscarriage after euploid blastocyst transfer in women with obesity have been reported [8,9], suggesting alternative mechanisms linking obesity to unfavorable reproductive outcomes. It should be considered, though, that not all women with obesity experience infertility or involuntary delay in spontaneous pregnancy [10], and only a proportion of women with overweight or obesity undergoing IVF have lower-than-expected outcomes [11]. One possible explanation for these heterogeneous findings could be the way overweight and obesity are commonly defined. Body mass index (BMI) is the most widely used parameter. Being based on weight and height, BMI represents a simple, inexpensive, and feasible method. According to the WHO classification, overweight is defined as a BMI between 25 kg/m^2^ and 29.9 kg/m^2^, whereas obesity is defined as a BMI greater than 30 kg/m^2^ [12]. Whilst the BMI-based classification is a very simple tool, some issues deserve consideration. Among these is the fact that BMI fails to identify nearly half of women with excess body fat [13,14], as it is not able to differentiate lean mass from fat mass. In addition to this limitation, most studies on BMI and reproduction do not distinguish between underweight and normal-weight women within the normal-weight BMI class, resulting in a potential risk of bias. More importantly, BMI does not measure body fat distribution. It has been known for some time that truncal adipose tissue (both visceral and subcutaneous) is positively associated with metabolic aberrations, such as insulin resistance and hyperinsulinemia, which are considered central features of the metabolic syndrome. As a matter of fact, the most recent literature points to altered metabolism and associated increased inflammatory response as major determinants of decreased reproductive potential in women, a correlation partially independent of BMI [15,16]. Given the strong association between central adiposity and insulin resistance, insulin sensitivity has emerged as a key metabolic factor potentially mediating the relationship between adiposity and reproductive outcomes. Impaired insulin sensitivity has been linked to anovulation, poor oocyte quality, and altered endometrial receptivity, all of which contribute to reduced fertility. Moreover, insulin resistance has been proposed to modulate ovarian responsiveness to gonadotropins, potentially affecting stimulation outcomes in ART cycles. Despite this, few studies have simultaneously evaluated body fat distribution and insulin sensitivity in the context of ART. The parameter of insulin sensitivity allows for a deeper understanding of the metabolic mechanisms that may explain the variability in ART response among women with similar anthropometric profiles [17]. Anthropometric measures and indexes other than BMI have been proposed to describe body fat distribution, such as waist circumference (WC), hip circumference, and waist-to-hip ratio (WHR). These measures and indexes have been found to predict metabolic risk better than BMI in various populations [18,19]. In particular, WC is currently considered a strong independent predictor of insulin resistance [20]. Given the suggested correlation between adiposity, metabolism, and reproduction, potential associations between central adiposity, fecundability, and time to pregnancy have also been investigated, resulting in non-univocal findings [21,22]. Early studies investigating the impact of body fat distribution on the outcome of in vitro fertilization (IVF) have reported conflicting results [23,24]. Since then, very few studies of the same kind have been published [25,26,27].

The aim of the present study was to assess the impact of increased body weight, abnormal fat distribution, and impaired insulin sensitivity on IVF/ICSI outcomes. Special attention was given to the ovarian sensitivity index (OSI) as a primary outcome measure in a cohort of women with infertility with normal or elevated BMI. This approach aims to better elucidate the metabolic factors contributing to variability in ART success beyond standard anthropometric classifications.

## 2. Methods

### 2.1. Study Design and Participants

This prospective observational study included 265 consecutive women undergoing their first homologous ART treatment (IVF/ICSI) between 2021 and 2023 at the Unit of Reproductive Medicine, S. Anna Hospital, University of Torino. The study was approved by the Ethics Committee of the University of Torino (Comitato Etico Interaziendale AOU Città della Salute e della Scienza di Torino), protocol number 0135216, approved on 10 June 2021. It adhered to the Declaration of Helsinki.

### 2.2. Inclusion and Exclusion Criteria

Inclusion criteria were as follows: age 18–44 years, regular menstrual cycles (25–32 days), basal (day 3) FSH < 20 IU/L, and AMH > 0.2 ng/mL. Exclusion criteria included BMI < 18.5 (underweight), polycystic ovary syndrome (PCOS; based on Rotterdam criteria [28]), systemic or metabolic diseases, medications affecting metabolism within 3 months prior to ART, prior pregnancies, oocyte/sperm donor cycles, and pre-implantation genetic testing.

### 2.3. Anthropometric and Biochemical Assessment

Before starting ovarian stimulation, anthropometric measurements and fasting blood samples were collected. Waist circumference (WC) and hip circumference (HC) were measured by the same operator according to WHO guidelines [19]. WC was measured at the midpoint between the lower rib margin and iliac crest; HC was measured around the widest part of the buttocks. Measurements were taken at end-expiration with participants in underwear or light clothing. Each measure was taken twice and averaged. The waist-to-hip ratio (WHR) was calculated as WC divided by HC. Fasting plasma glucose and insulin levels were assessed. The homeostatic model assessment (HOMA-IR) was used to evaluate insulin sensitivity using the formula: [(fasting glucose in mg/dL × fasting insulin in μIU/mL)/405] [29].

### 2.4. Controlled Ovarian Stimulation Protocols

Ovarian stimulation involved either a GnRH antagonist (Orgalutran, MSD, Rahway, NJ, USA) starting on day 5 of stimulation or a luteal-phase GnRH agonist (Suprefact, Sanofi-Aventis Canada Inc., or Decapeptyl, Ipsen, Paris, France). Gonadotropin stimulation consisted of recombinant FSH (Gonal F, Merck-Serono, Darmstadt, Germany), recombinant FSH + LH (Pergoveris, Merck-Serono, Germany), or menotropins (Meropur, Ferring, Saint Prex, Switzerland or Meriofert, IBSA) at a starting dose of 150–300 IU/day, based on a previously validated nomogram [30]. Monitoring included serial transvaginal ultrasounds and serum estradiol levels from stimulation days 6–7. Dosage was adjusted as necessary. Final oocyte maturation was triggered with 10,000 IU of hCG (Gonasi, IBSA, Switzerland), administered approximately 35 h before oocyte retrieval.

### 2.5. Oocyte Retrieval, Fertilization, and Embryo Transfer

Transvaginal ultrasound-guided oocyte pick-up was performed under local anesthesia. Mature oocytes (MII) were fertilized via conventional IVF or ICSI depending on semen quality. Fertilization was assessed 16–18 h later by the presence of two pronuclei (2PN).

Embryos were cultured and transferred either on day 3 (two top-quality embryos) or on day 5 (single expanded blastocyst), based on oocyte number and embryo quality. Embryo morphology was assessed using the Integrated Morphology Cleavage Score on day 2 [31] and the Istanbul Consensus criteria on day 5 [32].

### 2.6. Luteal Support and Pregnancy Evaluation

Luteal phase support consisted of 180 mg/day natural progesterone (Crinone 8%, Merck-Serono, Germany). Pregnancy was initially assessed via serum hCG 15 days post-ET and confirmed by ultrasound visualization of a gestational sac two weeks later. Only confirmed clinical pregnancies were counted; biochemical pregnancies were excluded. Live birth was defined as delivery after 24 weeks of gestation.

### 2.7. Study Groups and Outcomes

Participants were stratified by WC (>80 cm vs. ≤80 cm), WHR (>0.85 vs. ≤0.85), and HOMA-IR (>2.5 vs. ≤2.5) based on established cutoffs [19,29,33]. The primary outcomes were the number of oocytes retrieved and ovarian sensitivity index (OSI), calculated as: [(number of retrieved oocytes/total gonadotropin dose) × 1000] [34]. Secondary outcomes included total gonadotropin dose, duration of stimulation, average FSH dose, peak estradiol, number of MII oocytes, clinical pregnancy rate (CPR), miscarriage rate (MR), and live birth rate (LBR).

### 2.8. Sample Size and Statistical Analysis

A sample size of 224 participants (112 per WC group) was calculated based on the ability to detect a difference of 1.5 in the ovarian sensitivity index (OSI) with 80% power and a significance level of α = 0.05. This sample size was deemed sufficient to provide reliable estimates for the primary outcome. Continuous variables were expressed as the median and interquartile range (IQR) to ensure appropriate representation of non-normally distributed data, and categorical variables were presented as counts and percentages. For comparisons, non-parametric tests (Mann–Whitney U-test) were applied to continuous variables, as the data did not meet the assumptions for parametric testing. Chi-square or Fisher’s exact tests were used for categorical variables. Spearman correlation was employed to assess associations for non-parametric data, and multivariable logistic regression was used to adjust for potential confounders. BMI, WC, WHR, and HOMA-IR were analyzed as both continuous and categorical variables, with appropriate justifications for each statistical method. Although AMH is inversely correlated with BMI and may partially mediate the impact of adiposity on ovarian response, it remains a key marker of ovarian reserve and is associated with stimulation outcomes. To account for inter-individual variability in baseline ovarian potential, AMH was included as a covariate in multivariable models. Statistical analysis was performed using JMP version number 12.0.1 (SAS Institute Inc., Cary, NC, USA). A *p*-value of ≤0.05 was considered statistically significant. Continuous variables were compared across BMI groups using Bonferroni-corrected pairwise comparisons. A Bonferroni-adjusted *p*-value of <0.05 was considered statistically significant.

## 3. Results

A total of 265 women were enrolled in the study. Baseline clinical characteristics and IVF outcomes for the whole group are shown in Table 1. The mean age (SD) was 35.8 (± 4.4) years. The mean (SD) BMI, WC, and WHR were 24.0 kg/m^2^ (±4.2), 79.1 cm (±10.8), 0.85 (± 0.09), respectively.

According to WHO classification [12], 170 patients had a normal weight (BMI 18.5-24.9), 64 women had overweight (BMI 25–29.9), and 31 had obesity (29 patients in the subgroup of class I obesity [BMI 30–34.9], 2 patients in class II obesity [BMI ≥ 35]). As for the treatment protocol, 51.7% of patients were treated with GnRH antagonist pituitary suppression, whilst in the remaining 48.3%, GnRH agonists were used. Conventional IVF and ICSI were performed in 38.8% and 61.2% of the cycles, respectively. The relative proportion of IVF/ICSI did not differ between the BMI, WC, and WHR subgroups. In three women (all normal weight, with no insulin resistance), no oocytes were retrieved at ovum pick up; in such cases, calculation of OSI was not possible. In 11 women, no embryos were obtained, with no statistical difference between subgroups of BMI, WC, WHR, and HOMA index. These cases were included because the study aimed to evaluate fertilization and embryo development outcomes across different populations, and their exclusion could have biased the assessment of early reproductive performance.

The participants were divided into subgroups to define increased upper body fat according to widely accepted thresholds for WHR and WC, i.e., 0.85 and 80 cm, respectively [19,35].

### 3.1. BMI

When comparing BMI subgroups between normal weight, overweight, and obese women, WC, WHR, and HOMA index showed significantly increasing values across the groups (Table 2). BMI did not show a significant impact on any of the other variables. The number of embryos transferred was comparable across BMI categories (normal weight, overweight, and obesity groups; *p* = NS). Pairwise comparisons using Bonferroni correction confirmed the absence of statistically significant differences between normal weight vs. overweight (adjusted *p* = 1.00), normal weight vs. obesity (adjusted *p* = 1.00), and overweight vs. obesity (adjusted *p* = 1.00). Moreover, no statistically significant differences were found among BMI groups for key stimulation outcomes (mean FSH dose, total FSH dose, estradiol peak, number of retrieved and mature oocytes, OSI) after Bonferroni correction. Similarly, miscarriage rates did not significantly differ across BMI categories.

### 3.2. Waist-to-Hip Ratio (WHR)

Among the study population, 119 women had a WHR of ≤0.85, while 146 women presented with a WHR of >0.85. Mean BMI significantly differed between these WHR subgroups. Notably, a substantial proportion (47.5%) of women with a normal BMI (<25) exhibited a WHR of >0.85, suggesting that WHR may capture central adiposity not reflected by BMI alone. Baseline characteristics and ART outcomes are summarized in Table 3. No significant differences were observed between groups in terms of mean age, AFC, or AMH levels. However, women with a WHR of >0.85 showed a significantly higher HOMA index, indicating greater insulin resistance.

Regarding ART outcomes, women with a higher WHR required significantly greater total and mean gonadotropin doses, as well as longer stimulation durations, despite comparable baseline ovarian reserve markers (AMH and AFC). This resulted in a markedly lower ovarian sensitivity index (OSI) in the WHR > 0.85 group. Additionally, a trend toward reduced oocyte yield was observed in this subgroup.

Multivariable models adjusted for BMI, HOMA index, AMH, infertility diagnosis (including PCOS and male factor infertility), type of fertilization (IVF vs. ICSI), and stimulation protocol (agonist vs. antagonist) confirmed the independent association of WHR with increased gonadotropin requirements, reduced oocyte yield, and lower OSI. Interestingly, a higher fertilization rate was seen in women with WHR ≤ 0.85, primarily within the ICSI subgroup; however, no significant differences were found in clinical pregnancy, live birth, or miscarriage rates across WHR groups. These findings underscore the relevance of central adiposity, beyond general obesity, in influencing ovarian responsiveness, and suggest that WHR may be a valuable, independent predictor in ART settings.

In 163 women, WC was ≤80 cm, whereas in 102 women, it was >80 cm. Mean BMI differed between the WHR subgroups. About 16% of the women with normal BMI (<25) had a WC of >0.85. Baseline characteristics and ART outcomes according to WC subgroups are reported in Table 4. AFC and AMH were similar between the groups. Conversely, the mean and total FSH doses were significantly different, and so were the HOMA index, the OSI, and the mean and total FSH doses. The differences in mean and total FSH dose, and in OSI, persisted even after adjusting for BMI HOMA index and AMH. In line with the findings for WHR, no differences were registered in clinical pregnancy, live birth rate, or miscarriage rate between the WC subgroups.

### 3.3. HOMA Index

According to a HOMA threshold > 2.50 [29], 30 women (11%) had insulin resistance. The only variable significantly different between women with insulin resistance and women with normal insulin sensitivity was BMI (29.9 + 2.9 vs. 23.3 + 3.7, respectively, *p* < 0.05). Of note, 65 women with overweight/obesity had normal insulin sensitivity. When managed as a continuous variable, the HOMA index showed a moderate degree of correlation with BMI (R^2^ 0.274, *p* < 0.05) and WC (R^2^ 0.223, *p* < 0.05), and a weak correlation with WHR (R^2^ 0.050, *p* < 0.05). No correlation was shown with any of the other variables investigated, with no significant differences between women with and without insulin resistance.

## 4. Discussion

The main reason prompting us to perform the present study is the fact that, whereas overweight and obesity are increasing steadily, the role played by these conditions in female fertility is still controversial [36]. It has been suggested that the type and disposition of body fat could play a more relevant role in human fertility than body mass per se [22]. As a matter of fact, according to WC and WHR, we observed a large variation in fat distribution among women within the same BMI categories. This finding confirms what has been shown in other series of infertile patients [25] and suggests that these indexes could provide different information on the clinical status of the individual. Whilst increased truncal-abdominal fat has been initially associated with both metabolic aberrations and decreased reproductive performance specifically in women with PCOS, recent studies suggest that abdominal obesity could be detrimental even in ovulatory women with normal ovaries [37]. In the present study, measures of body fat distribution were investigated in relation to the outcome of a first IVF/ICSI cycle in a series of women accurately defined as non-PCOS, distributed across a wide range of BMI. The first conclusion that can be drawn from the present results is that, whereas BMI correlates positively, as expected, with WC and WHR, those measures of body fat distribution perform better in predicting ovarian sensitivity to gonadotropin stimulation. Indeed, both WC and WHR were associated with the dose of gonadotropins administered during ovarian stimulation (positively) and ovarian sensitivity, as assessed by OSI (inversely). Of note, the association was independent not only from BMI but also from the HOMA index. As a matter of fact, women deemed insulin-resistant, according to a widely used threshold [29], did not differ from those with normal insulin sensitivity in any variable associated with ovarian stimulation/sensitivity. Contrasting this finding, a recent retrospective cohort study reported a negative association between the HOMA index and OSI, with a potentially stronger correlation among women with PCOS and women with overweight/obesity [38]. The authors discussed the results, pointing at hyperinsulinemia as the main cause of altered ovarian physiology and of reduced response to exogenous gonadotropins. Indeed, independent groups have previously shown such a correlation in women with PCOS [39,40], suggesting that it could be an intrinsic feature of the syndrome. It is a common notion that these patients are particularly prone to accumulating upper-body fat, with consequences on metabolism and ovarian function that are far more clinically relevant than in the non-PCOS population [41]. Taking into account all the above evidence, one possible explanation for the discrepancy between the present results and those of the mentioned study [38] might lie in the different compositions of the study groups. About 20% of the ART cycles in the large retrospective study were performed in women with PCOS [38]. A second possible explanation for the lack of correlation between HOMA and OSI could be that the HOMA index, a very simple and rather inexpensive method for measuring insulin sensitivity, might not be as accurate as more sophisticated tests [42]. On the other hand, whereas more efficient diagnostic procedures, such as the euglycemic clamp, could be used, only simple tests should qualify for routine use in clinical settings. Nevertheless, future research could benefit from the adoption of more complex and accurate methods of insulin resistance assessment, such as the euglycemic clamp, which is considered the gold standard technique for quantifying insulin sensitivity [43]. Although its application in clinical practice is limited by technical complexity, high cost, and time consumption, its use in dedicated research settings could allow a better characterization of subtle metabolic alterations potentially impacting ovarian physiology. Furthermore, combining the clamp technique with dynamic assessments of inflammatory and oxidative stress markers could shed new light on the biological pathways linking central adiposity to reproductive function. Finally, the HOMA threshold for the definition of insulin resistance, which varies between studies [38,39], could also be an explanation for the differing results. However, whereas the limit used for defining insulin resistance in the present series of patients might be considered somewhat arbitrary, a sub-analysis comparing OSI according to tertiles of the HOMA distribution failed to find differences in ovarian sensitivity. A recent large retrospective study on 1104 non-PCOS women showed that although both insulin resistance (as assessed by the HOMA index) and hyperinsulinemia were associated with altered hormonal milieu, no impact was observed on either pregnancy rates or miscarriage rates [44]. Although one limitation of the study was the restricted distribution of BMI (most women were reportedly lean), the results are in line with the present findings. Thus, our results and others’ [44] point to mechanisms other than insulin resistance, linking truncal abdominal fat to ovarian function, even in non-PCOS women. Body fat is not only related to insulin resistance, but it constitutes a source of inflammatory molecules, which are implicated in increased oxidative stress [45]. Furthermore, central obesity seems to account for specific profiles of cytokine production [46]. In this regard, chronic low-grade inflammation may negatively influence follicular microenvironment, oocyte competence, and endometrial receptivity. Similarly, enhanced oxidative stress observed in abdominal obesity could impair mitochondrial function in the oocyte, with consequences on fertilization and embryo quality. Exploring these biological pathways could open new perspectives for targeted therapeutic interventions aimed at improving ART outcomes in women with central adiposity. In line with this hypothesis, abdominal obesity, as measured by WHR, has been associated with reduced antioxidant activity within follicular fluid in both women with PCOS and normal controls [47]. Whatever the mechanism linking abdominal fat to an altered follicular dynamic, the putative impact of upper body obesity on oocyte quantity and quality, according to the present results, seems to be of low relevance. Indeed, neither the total number nor the proportion of mature oocytes (MII) differed according to WC classification, although a slightly lower proportion of fertilized oocytes was observed in the group of women with higher WHR. The fact that the difference was limited to the subgroup of ICSI cycles suggests that factors not necessarily implicated in fat distribution might have influenced fertilization in the present study. These results contrast with the findings of a recent cross-sectional observational study [25] performed on a large series of infertile women distributed across a wide range of BMIs, which found negative and significant associations between the anthropometric indicators (BMI, WC, and WHR) and the number of total and MII oocytes retrieved. However, it should be considered that no index of ovarian sensitivity to gonadotropins was reported in the cited study [25]. Hence, it is not possible to know whether the number of oocytes recovered would, in fact, depend on the dose of gonadotropins administered. The OSI, which adjusts the ovarian response for the exogenous FSH dose, might be a more accurate indicator of follicular dynamics than the number of retrieved oocytes per se [48,49]. Furthermore, in the study by Christofolini et al. [25], measures of body fat distribution were associated with the probability of cycle cancellation (due to a lack of response), whereas no cycles were canceled before oocyte retrieval in the present series. This could either denote differences in the study groups or a different clinical approach (e.g., different thresholds for defining lack of response and/or different doses of gonadotropins administered). Whatever the mechanism, the results point to a role of body fat distribution in follicular dynamics, at least under supra-physiological stimulation such as that applied during ART. In our study, the secondary outcomes, i.e., the clinical pregnancy rate, the live birth rate, and the miscarriage rate, did not differ between subgroups. Few studies so far have investigated the relationship between anthropometric measures and ART outcomes in terms of pregnancy rates. An early study from Scandinavia showed that an android fat distribution (WHR > 0.80) was negatively associated with the clinical pregnancy rate in IVF cycles, even after adjusting for age, BMI, and other possible confounders [24]. Although the authors adjusted for the number of embryos transferred in utero, no indications of ovarian reserve, response to ovarian stimulation, or insulin sensitivity were reported. Furthermore, pregnancy up to 4–5 weeks after embryo transfer was considered the primary endpoint, whereas no data on MR and LBR were available. Recently, a prospective study from North America reported a significant, progressive decline in pregnancy rates and live births with increasing values of WC [26]. Interestingly, women with overweight with normal WC had similar outcomes to women with a normal weight, strongly pointing to a detrimental role of abdominal fat on ART outcome. Since the declining proportion of live births reflected a progressive decline in implantation rates, but not in fertilization rates, with increasing WC, a potential role of the endometrium receptivity could be considered. However, no data on pregnancy loss were reported. Whereas the results in terms of clinical pregnancies and live births are at variance with those from the present investigation, the designs of the studies differ in several aspects. Some of the women enrolled in the study by Li et al. [26] underwent repeated ART cycles (445 cycles were performed in 264 women), whereas only the first cycles were included in our study. Importantly, the North American study included both conventional and egg donor cycles, along with both fresh and cryo/thaw cycles, which did not allow for an appropriate comparison. On the other hand, the lack of any impact of body fat distribution on the clinical outcomes of ART shown by our results corroborates the findings of some recent reports. An intervention study performed in women predominantly with overweight and obesity undergoing a weight-reduction program failed to show any correlation between BMI or WC and pregnancy or live birth rates after IVF [50]. Another large retrospective study on 1134 ART cycles comparing women who conceived versus those who did not become pregnant found that, although a positive outcome was associated with lower BMI, no differences existed between the study groups in terms of WHR [23]. Finally, a prospective observational study including 402 couples undergoing the ICSI cycle failed to demonstrate a significant negative impact of WC and WHR on both pregnancy and miscarriage rates [27]. The strengths of our study are its prospective nature and the fact that all anthropometric measurements were taken by only one trained operator, following a strict and standardized method [19], thus avoiding the risk of inter-observer variability. Furthermore, only couples undergoing their first ART cycle were consecutively recruited, thus avoiding the confounding impact of repeated non-independent cycles. Finally, underweight women were not included in the study group. One limitation of this study is the sample size and the unequal distribution of participants across BMI categories. Only a few patients with BMI ≥ 30 kg/m^2^ were enrolled. One reason for this underrepresentation of women with obesity is the current local recommendation to discourage pregnancy in this subgroup, advising weight loss and BMI optimization before starting ovarian stimulation for ART. Additionally, the exclusion of underweight women (BMI < 18.5 kg/m^2^) limits the interpretation of our findings across the full spectrum of body weight. While this decision aimed to reduce potential confounding effects related to malnutrition or other underlying conditions, it may have also excluded a subset of the infertile population with distinct reproductive profiles. Therefore, while our results are applicable to women with normal to overweight BMI, caution should be exercised when extrapolating these findings to individuals with underweight and obesity. A further limitation of the study is that only fresh embryo transfers were included in the analysis. Consequently, it is not possible to determine whether outcomes from frozen/thawed embryo transfers would vary according to body mass and fat distribution. Future studies integrating frozen embryo transfer (FET) outcomes would be particularly valuable, as they could provide additional insights into whether endometrial receptivity or embryo competence is primarily affected by fat distribution patterns. However, the number of vitrified blastocysts did not differ between the BMI, WHR, and WC subgroups. Moreover, recent data suggest that live birth rates are not adversely affected by obesity in the context of frozen/thawed blastocyst transfers [51].

## 5. Conclusions

In conclusion, the results of our study on infertile women undergoing their first ART cycle, reinforce the notion that the type of body fat distribution, which is largely independent from total body mass, is associated with the ovarian response to pharmacological stimulation. In particular, upper body fat correlates negatively with ovarian sensitivity to exogenous gonadotropins. Although these findings may not translate into differences in fertilization, pregnancy, or live birth rates after the first embryo transfer, they highlight the importance of considering fat distribution, and not merely BMI, in the counseling and clinical management of women undergoing ART. Tailored interventions aimed at reducing central adiposity, rather than weight loss per se, could potentially optimize ovarian responsiveness and improve overall reproductive outcomes. The mechanisms, beyond a state of insulin resistance, that are implicated in this phenomenon remain to be determined. Biological pathways such as chronic inflammation and oxidative stress are plausible candidates and should be explored in future investigations. Moreover, extending the analysis to include frozen/thawed embryo transfer outcomes could broaden our understanding of how fat distribution affects endometrial and embryonic factors, contributing to the success of ART treatments [39,46].

## Figures and Tables

**Table 1:** Baseline characteristics of the study population. Continuous variables are reported as mean + SD when normally distributed (according to Shapiro–Wilk test for normality) and as median [interquartile range] otherwise.

Number of participants	265
Age (years)	36 [33–39]
BMI (kg/m^2^)	22.8 [20.9–26.9]
Smoker, n (%)	
Never	67%
Past	12%
Current	21%
Infertility diagnosis	
Male factor	40.7% (108/265)
Female factor	39.6% (105/265)
Unexplained	19.6% (52/265)
Treatment protocol	
GnRH antagonist	52.0% (138/265)
GnRH agonist	48.0% (127/265)
IVF	38.8% (103/265)
ICSI	61.2% (162/265)
Hip circumference (HC)	92.8 ± 10.5
Waist circumference (WC)	86 [75–96]
Waist-to-hip ratio (WHR), mean (SD)	0.93 [0.86–1.2]
AFC	12 [8–20]
AMH	2.4 [1.1–4.9]
HOMA index	1.06 [0.59–1.81]

BMI = Body Mass Index; GnRH = Gonadotropin-Releasing Hormone; IVF = In Vitro Fertilization; ICSI = Intracytoplasmic Sperm Injection; HC = Hip Circumference; WC = Waist Circumference; WHR = Waist-to-Hip Ratio; AFC = Antral Follicle Count; AMH = Anti-Müllerian Hormone; SD = Standard Deviation.

**Table 2:** Baseline characteristics and ART outcomes according to BMI. Continuous variables reported as mean ± SD.

	BMI < 25	BMI ≥ 25–29.9	BMI ≥ 30	*p*-Value
N	170	64	31	
Age (years)	36 ± 3.9	35.8 ± 5.0	34.8 ± 5.4	NS
WC (cm)	73.8 ± 6.9	85.4 ± 3.7	95.8 ± 8.9	<0.05
WHR	0.84 ± 0.09	0.86 ± 0.08	0.9 ± 0.07	<0.05
AFC	14.7 ± 9.5	14.6 ± 9.3	12.9 ± 8.4	NS
AMH (ƿg/L).	3.67 ± 4.42	3.62 ± 4.22	3.22 ± 2.90	NS
HOMA index.	1.00 ± 0.91	2.21 ± 1.81	2.51 ± 1.88	<0.05
Mean FSH dose (UI).	216 ± 85	229 ± 83	228 ± 86	NS
Total FSH dose (UI).	2451 ± 1189	2706 ± 1249	2686 ± 1030	NS
Duration of stimulation (days)	11.2 ± 1.9	11.7 ± 2.2	11.7 ± 1.8	NS
Estradiol peak at trigger (pmol/L)	1728 ± 1227	1678 ± 1080	1440 ± 699	NS
Number of retrieved oocytes	8.9 ± 6.1	8.0 ± 5.7	8.2 ± 5.2	NS
Number of mature oocytes (MII)	7.2 ± 4.7	6.1 ± 4.2	6.8 ± 4.7	NS
OSI	5.2 ± 5.3	4.2 ± 4.6	3.6 ± 2.9	NS
Fertilization rate	85.8 ± 21.1	85.4 ± 24.9	82.1 ± 27.2	NS
Fertilization rate				
IVF	90 ± 14	97 ± 6	94 ± 12	NS
ICSI	82 ± 24	81 ± 28	78 ± 30	NS
Proportion ICSI/IVF (%)	68.2/31.8	73.4/26.6	74.2/25.8	NS
Number of embryos to ET	1.6 ± 0.5	1.5 ± 0.5	1.7 ± 0.5	NS
Proportion D3/D5 to ET (%)	69.7/30.3	70.8/29.2	76.9/23.1	NS
Cryopreserved blastocysts	1.7 ± 1.2	1.6 ± 1.1	1.9 ± 1.5	NS
Clinical pregnancy rate *	41% (65/159)	41% (23/56)	26% (7/27)	NS
Miscarriage rate *	15% (10/65)	5% (3/56)	7% (2/27)	NS
Live birth rate *	35% (55/159)	36% (20/56)	19% (5/27)	NS

* Data on pregnancy outcome were available for 242 patients. BMI = Body Mass Index; WC = Waist Circumference; WHR = Waist-to-Hip Ratio; AFC = Antral Follicle Count; AMH = Anti-Müllerian Hormone; HOMA = Homeostasis Model Assessment; FSH = Follicle-Stimulating Hormone; OSI = Ovarian Sensitivity Index; ICSI = Intracytoplasmic Sperm Injection; IVF = In Vitro Fertilization; ET = Embryo Transfer; D3/D5 = Day 3/Day 5; SD = Standard Deviation; NS = Not Significant.

**Table 3:** Baseline characteristics and ART outcomes in WHR subgroups. Continuous variables reported as mean ± SD.

	WHR ≥ 0.85	WHR < 0.85	*p*-Value
N	146	119	
Age (years)	36 ± 4.5	35.6 ± 4.3	NS
BMI (kg/m^2^)	24.7 ± 4.5	23.2 ± 3.7	<0.05
AFC	14.6 ± 9.2	14.4 ± 9.4	NS
AMH (ƿg/L)	3.67 ± 4.48	3.47 ± 3.82	NS
HOMA index	1.54 ± 1.50	1.16 ± 1.05	<0.05
Mean FSH dose (UI)	232 ± 80	204 ± 87	<0.05
Total FSH dose (UI)	2622 ± 1060	2423 ± 1321	<0.05
Duration of stimulation (days)	11 ± 1.7	11 ± 2.2	<0.05
Estradiol peak at trigger (pmol/L)	1736 ± 1253	1911 ± 984	NS
Number of retrieved oocytes	7.3 ± 4.2	8.8 ± 5.6	NS
Number of mature oocytes (MII)	6.0 ± 3.7	7.1 ± 4.6	NS
OSI	3.4 ± 2.3	4.9 ± 4.1	<0.05
Fertilization rate	82.9 ± 24.0	88.3 ± 20.7	<0.05
Fertilization rate			
IVF	92 ± 13	91 ± 13	NS
ICSI	78 ± 27	86 ± 24	<0.01
Proportion ICSI/IVF (%)	65.7/34.3	61.3/38.7	NS
Number of embryos to ET	1.5 ± 0.5	1.6 ± 0.5	NS
Proportion D3/D5 to ET (%)	67.5/32.5	74.5/25.5	NS
Cryopreserved blastocysts	1.6 ± 1.3	1.7 ± 1.1	NS
Clinical pregnancy rate *	39% (53/134)	38.5% (42/108)	NS
Miscarriage rate *	19% (10/53)	12% (5/42)	NS
Live birth rate *	32% (43/134)	34% (37/108)	NS

* Data on pregnancy outcome were available for 242 patients. Waist circumference (WC) WHR = Waist-to-Hip Ratio; BMI = Body Mass Index; AFC = Antral Follicle Count; AMH = Anti-Müllerian Hormone; HOMA = Homeostasis Model Assessment; FSH = Follicle-Stimulating Hormone; OSI = Ovarian Sensitivity Index; IVF = In Vitro Fertilization; ICSI = Intracytoplasmic Sperm Injection; ET = Embryo Transfer; D3/D5 = Day 3/Day 5; SD = Standard Deviation; NS = Not Significant.

**Table 4:** Baseline characteristics and ART outcomes in WC subgroups.

	WC > 80 cm	WC ≤ 80 cm	*p*-Value
N	102	163	
Age (years)	35.7 ± 4.6	35.9 ± 4.1	NS
BMI (kg/m^2^)	27.7 ± 3.8	21.8 ± 2.6	<0.05
AFC	13.6 ± 8.5	15.1 ± 9.7	NS
AMH (ƿg/L)	3.0 ± 2.6	3.9 ± 4.8	NS
HOMA index	2.1 ± 1.8	0.9 ± 0.6	<0.05
Mean FSH dose (UI)	232 ± 78	212 ± 87	<0.05
Total FSH dosage (UI)	2662 ± 1069	2452 ± 1249	<0.05
Duration of stimulation (days)	11 ± 2	11 ± 1.9	NS
Estradiol peak at trigger (pmol/L)	1732 ± 1294	1648 ± 1036	NS
Number of retrieved oocytes	7.1 ± 4.5	8.4 ± 4.5	NS
Number of mature oocytes (MII)	6.0 ± 3.9	6.8 ± 4.3	NS
OSI	3.2 ± 2.5	4.6 ± 3.9	<0.05
Fertilization rate	84.2 ± 22.8	86.0 ± 22.7	NS
Proportion ICSI/IVF (%)	69.6/30.4	60.1/39.9	NS
Number of embryos to ET	1.6 ± 0.5	1.6 ± 0.5	NS
Proportion D3/D5 to ET (%)	69.1/30.9	71.7/28.3	NS
Cryopreserved blastocysts	1.6 ± 1.2	1.7 ± 1.2	NS
Clinical pregnancy rate *	39% (36/91)	39% (59/151)	NS
Miscarriage rate *	19% (7/36)	14% (8/59)	NS
Live birth rate *	32% (29/92)	34% (51/152)	NS

* Data on pregnancy outcome were available for 242 patients. WC = Waist Circumference; BMI = Body Mass Index; AFC = Antral Follicle Count; AMH = Anti-Müllerian Hormone; HOMA = Homeostasis Model Assessment; FSH = Follicle-Stimulating Hormone; OSI = Ovarian Sensitivity Index; ICSI = Intracytoplasmic Sperm Injection; IVF = In Vitro Fertilization; ET = Embryo Transfer; D3/D5 = Day 3/Day 5; SD = Standard Deviation; NS = Not Significant.

## Data Availability

The datasets used and/or analyzed in the current study are available from the corresponding author upon reasonable request.

## Abbreviations

Assisted reproductive technologies (ARTs), preimplantation genetic testing (PGT), in vitro fertilization (IVF), World Health Organization (WHO), body mass index (BMI), waist circumference (WC), hip circumference (HC), waist-to-hip ratio (WHR), follicle-stimulating hormone (FSH), anti-Müllerian hormone (AMH), polycystic ovary syndrome (PCOS), homeostatic model assessment (HOMA), gonadotropin-releasing hormone (GnRH), follicle-stimulating hormone (FSH), luteinizing hormone (LH), transvaginal ultrasound (TV-US).
